# Iterative nanoparticle bioengineering enabled by x-ray fluorescence imaging

**DOI:** 10.1126/sciadv.adl2267

**Published:** 2024-03-22

**Authors:** Giovanni M. Saladino, Bertha Brodin, Ronak Kakadiya, Muhammet S. Toprak, Hans M. Hertz

**Affiliations:** Department of Applied Physics, Biomedical and X-Ray Physics, KTH Royal Institute of Technology, SE 10691, Stockholm, Sweden.

## Abstract

Nanoparticles (NPs) are currently developed for drug delivery and molecular imaging. However, they often get intercepted before reaching their target, leading to low targeting efficacy and signal-to-noise ratio. They tend to accumulate in organs like lungs, liver, kidneys, and spleen. The remedy is to iteratively engineer NP surface properties and administration strategies, presently a time-consuming process that includes organ dissection at different time points. To improve this, we propose a rapid iterative approach using whole-animal x-ray fluorescence (XRF) imaging to systematically evaluate NP distribution in vivo. We applied this method to molybdenum-based NPs and clodronate liposomes for tumor targeting with transient macrophage depletion, leading to reduced accumulations in lungs and liver and eventual tumor detection. XRF computed tomography (XFCT) provided 3D insight into NP distribution within the tumor. We validated the results using a multiscale imaging approach with dye-doped NPs and gene expression analysis for nanotoxicological profiling. XRF imaging holds potential for advancing therapeutics and diagnostics in preclinical pharmacokinetic studies.

## Teaser

X-ray fluorescence imaging for iterative nanoparticle bioengineering tackles nanomedicine challenges in targeting efficiency.

## INTRODUCTION

Iterative bioengineering plays a crucial role in the development and optimization of nanoparticles (NPs) for various biomedical applications, particularly in the field of nanomedicine (*1*–*3*). It refers to a continuous feedback loop of NP design, synthesis, characterization, and preclinical testing. The design of contrast agents for preclinical and clinical imaging must take into consideration NP characteristics including composition, size, and shape, which play a substantial role in tuning biodistribution and toxicity (*4*). NP properties, such as surface chemistry, wettability, and surface charge have been shown to influence the extent of interaction of nanomaterials with biological systems, affecting the composition of the protein corona, the NP cellular uptake, physiological response, and clearance (*1*, *5*, *6*).

Undesired NP accumulations within organs, such as the liver and lungs, pose a major challenge in the field of nanomedicine. While NPs hold a high potential for improved diagnosis and therapy, their unintended sequestration can result in adverse effects, potentially leading to compromised organ function and inflammation, thus limiting their overall applicability (*7*). Furthermore, recent studies have demonstrated that less than 1% of administered NPs are successfully delivered to solid tumors, due to NP sequestration by the mononuclear phagocytic system (*8*), contributing to low clinical translation rates (*9*, *10*). For this reason, the evaluation of NP toxicity in vitro using different cell assays is necessary but not sufficient to predict potential toxic effects in vivo. In this respect, NP design and optimization processes go through several preclinical iterations in vivo before achieving the desired results, thus constituting a major barrier toward the optimization of nanomedicines. Within these circumstances, it is necessary to develop methods that allow fast and efficient preclinical studies, aiming at gathering information about NP biodistribution, retention in major organs, and excretion to improve targeting efficiency and minimize adverse effects, eventually facilitating clinical translation (*11*, *12*).

Typically, in vivo preclinical pharmacokinetics uses classical imaging techniques, such as magnetic resonance imaging (MRI), positron emission tomography, and optical imaging. These methods suffer from low specificity, radiolabeling requirement, and low penetration depth, respectively (*1*, *13*, *14*). Furthermore, time-consuming histological analysis is often required to confirm the NP localization and distribution within tissues, to verify targeting efficacy and to perform comparative investigations (*15*).

X-ray fluorescence (XRF) has recently been used for in vivo imaging of small animals with high elemental specificity, by designing inorganic NPs as contrast agents, composed of elements whose absorption edge matches the x-ray source energy (24 keV), such as molybdenum (Mo), ruthenium (Ru), or rhodium (Rh) (*16*–*20*). In our previous study, the formation of a core-shell hybrid nanostructure, with an XRF active core (MoO_2_ NPs) and a passivating dye-doped silica (SiO_2_) shell, enabled dual optical and XRF imaging and reduced the cytotoxicity in vitro, as compared to uncoated MoO_2_ NPs (*18*). In the present work, we pursue the possibility of assessing the NP performance through iterative bioengineering, allowing the rapid identification of improvement options on the NP design to generate more efficient solutions and functionalities.

## RESULTS

### Concept and iterative approach

**Fig. 1.**
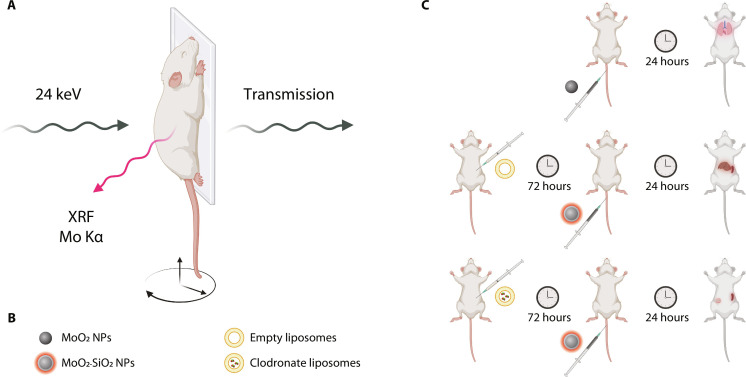
Iterative bioengineering approach. (**A**) Schematic illustration of XRF imaging, highlighting the x-ray source energy, transmitted photons, XRF photons, and movable stage. (**B**) Representation of the four NP types used for the iterative study: core MoO_2_ NPs, fluorescent core-shell MoO_2_-SiO_2_ NPs, empty liposomes, and clodronate-encapsulated liposomes. (**C**) In vivo methodology for XRF imaging, leading to the reduction of unwanted accumulations in lungs and liver and the eventual tumor detection. Portions of this figure were created using BioRender.com.

We conceptually illustrated the iterative bioengineering approach with XRF imaging (Fig. 1). A schematic representation of the in vivo XRF imaging setup (Fig. 1A) highlighted the employment of a focused x-ray beam of 24 keV, generating XRF radiation when exciting an element with matching adsorption edge to the x-ray source energy (*21*). The unabsorbed radiation was used for conventional x-ray (transmission) imaging as the spatial reference for the XRF signal. An anesthetized mouse was placed on a vertical holder, which was moved in the *x*-*y* direction to acquire a projection image, while the stage could be rotated to generate an x-ray fluorescence tomography (XFCT). Mo-based NPs were chosen as the contrast agent for this study. MoO_2_ NPs and MoO_2_-SiO_2_ NPs were studied to evaluate their biodistribution via XRF imaging and assess their biocompatibility. Furthermore, to circumvent the major role of the hepatic macrophages (Kupffer cells) in NP sequestration (*8*), empty liposomes and clodronate-encapsulated liposomes were pre-injected to investigate the effect of transient Kupffer cell depletion on the organ distribution and on the possibility of tumor detection enabled by higher NP accumulations owing to an increased circulation time (Fig. 1B). XRF imaging was used as a tool for iterative bioengineering of NPs as contrast agents. The in vivo biodistribution study enabled the advancement of NP optimization and the selection of the best approach to achieve passive tumor targeting and prevent undesired accumulations in organs such as lungs and liver (Fig. 1C).

### Contrast agent design

**Fig. 2.**
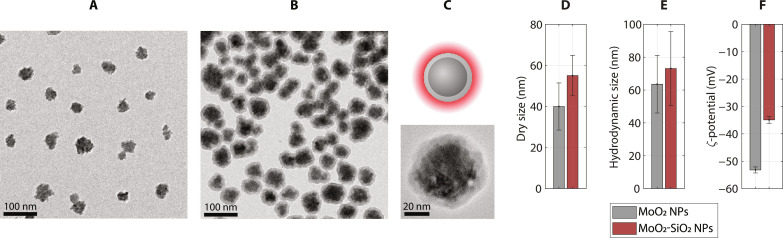
Nanoparticle characterization. TEM images of (**A**) core MoO_2_ NPs and (**B**) core-shell MoO_2_-SiO_2_ NPs. (**C**) Schematic representation and magnified TEM micrograph of a single core-shell NP. (**D**) Dry size (TEM), (**E**) hydrodynamic size, and (**F**) ζ-potential of MoO_2_ NPs (gray) and MoO_2_-SiO_2_ NPs (red).

MoO_2_ NPs were synthesized through a solvothermal method, using polyvinylpyrrolidone (PVP) as the capping agent, resulting in small spherical clusters of 40 ± 12 nm in diameter, as observed with transmission electron microscopy (TEM) (Fig. 2, A and D). A thin silica shell was condensed on the surface of MoO_2_ NPs via a modified Stöber method, using Cy5.5 as the doping fluorophore. A schematic representation of the two-step synthesis route was presented (fig. S1). The use of lower amounts of silica precursor yielded thinner shells than previously reported (*18*). The obtained MoO_2_-SiO_2_ NPs exhibited a uniform coating (Fig. 2, B and C), with an overall average diameter (dry size) of 55 ± 10 nm (Fig. 2D). The acquired values for polydispersity index (PDI) could also provide a measure of NP size homogeneity. The hydrodynamic size followed a similar trend, with values equal to 64 ± 18 nm (PDI, 0.12) and 73 ± 23 nm (PDI, 0.19) for MoO_2_ NPs and MoO_2_-SiO_2_ NPs, respectively. Together with low PDIs, the strongly negative ζ-potential values (<−30 mV) confirmed the NP stability and low polydispersity in aqueous media (Fig. 2F), as the major requirements for biomedical applications (*22*, *23*). Selected area diffraction (SAED) with TEM (fig. S2A) provided information about the crystal structure of MoO_2_ NPs, after silica coating: MoO_2_-SiO_2_ NPs exhibited the diffraction pattern (fig. S2B) of MoO_2_ with hexagonal crystal structure [International Centre for Diffraction Data (ICDD) card no. 00-050-0739], like the as-synthesized MoO_2_ NPs (*18*, *24*), demonstrating that the modified Stöber method did not affect the core NP structure. Ethanolamine (EA) permits a faster reaction rate, due to its strong catalytic effect (*18*, *25*), thus limiting the exposure of the core MoO_2_ NPs. The silica formation was confirmed with Fourier transform infrared (FTIR) spectroscopy, where the Si─O─Si stretching vibration at 1079 cm^−1^ was evidenced (fig. S3A) (*26*). Furthermore, the band at 1637 cm^−1^ ascribed to C═O stretching confirmed the presence of chemisorbed PVP via the carbonyl group, demonstrated by the shift from the value obtained for free PVP in water (1632 cm^−1^). It is, moreover, an indication of no molecular replacement on the core surface and the silica growth on PVP. The optical fluorescence properties of MoO_2_-SiO_2_ NPs were confirmed by photoluminescence (PL) excitation and emission spectra, with peaks at 672 and 686 nm, respectively (fig. S3B). These spectra demonstrated the successful Cy5.5 doping of the silica shell. The synthesized MoO_2_-SiO_2_ NPs were evaluated in vitro for their cytotoxicity using two cell lines, evidencing a concentration-dependent viability (fig. S4, A and B). Further details were provided in the Supplementary Materials.

### XRF imaging and iterative bioengineering

**Fig. 3.**
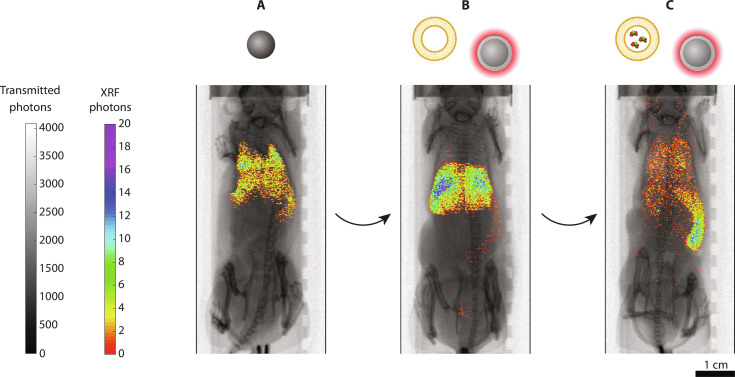
In vivo XRF imaging. XRF projection images of mice injected with (**A**) MoO_2_ NPs, (**B**) empty liposomes and MoO_2_-SiO_2_ NPs, and (**C**) clodronate-encapsulated liposomes and MoO_2_-SiO_2_ NPs. XRF signal (color) was overlaid on top of x-ray absorption (grayscale). Liposomes were injected 72 hours before MoO_2_-SiO_2_ NP injection. Images were acquired 1 hour after NP injection. Scale bar, 1 cm. Portions of this figure were created using BioRender.com.

We demonstrated the role of XRF imaging for our iterative bioengineering process, by using MoO_2_ NPs and MoO_2_-SiO_2_ NPs. Imaging was performed 1 hour after NP injection. MoO_2_ NPs were evaluated with a prestudy to confirm the previously observed results (*27*): They mainly accumulated in the lungs and liver, as could be visualized in the projection image, where the XRF signal (Mo Kα) followed the lung morphology within the thoracic cavity of the chest (Fig. 3A and fig. S5). The XRF integrated signal decreased over time, ascribed to a probable NP clearance from the body. Minor signal was detected in the spleen too.

The second iteration consisted of the introduction of a dye-doped silica shell, yielding MoO_2_-SiO_2_ NPs. Mice injected with MoO_2_-SiO_2_ NPs exhibited a different biodistribution with XRF imaging (Fig. 3B and fig. S6). After the injection (1 hour), XRF projection imaging led to the predominant detection of XRF signal in the liver, reported as the main NP clearance pathway for these NP size ranges (*28*, *29*). The overall biodistribution did not substantially vary over 24 hours, with only a decrease in the XRF signal from the liver, indicating NP excretion by the hepatobiliary system (*28*). Consistent results were observed with the four injected mice (fig. S6), with fluctuations only in the XRF intensity ascribed to random errors during the injection process. Occasionally, XRF photons were also detected from the bladder.

The XRF projection images on mice injected with clodronate-encapsulated liposomes and MoO_2_-SiO_2_ NPs provided information on the extent of the effect of clodronate on the liver and, consequently, the overall biodistribution (Fig. 3C and fig. S7). Noticeably, scattered XRF signal was detected throughout the whole body, including the largest vasculature supplying the ophthalmic artery and the cerebral tissue, among others. Although some signal could be detected in lungs and liver, most of the signal (>70%) was localized in the spleen. Furthermore, signs of splenomegaly were also observed in all the imaged mice. Extracted spleens from both controls and injected mice exhibited similar sizes (fig. S8A), with the largest dimension exceeding 2 cm. This observation led to the conclusion that the spleen enlargement was induced by the xenografted 4T1 cells, known to provoke a leukemoid reaction with splenomegaly in BALB/c mice (*30*). As a comparison, mice which did not undergo tumor xenografting featured a smaller spleen (≈1 cm), indirectly observable through the detected XRF photons (Mo Kα) following its shape (fig. S5).

### In vivo tumor detection

BALB/c mice were xenografted with the syngeneic breast tumor adenocarcinoma cell line 4T1. This tumor type is characterized by quick local growth and metastatic spread within 3 to 4 weeks (*31*). Our focus was the primary solid tumor detection, which exhibited an exponential growth (*R*^2^ = 0.9913), measured with palpation and followed by ellipsoid approximation (fig. S9A), confirming the growth rate analysis of previous studies (*32*). Mice were injected with clodronate-encapsulated liposomes within 13 days from the tumor xenograft; tumors reached a final volume of at least 400 mm^3^, corresponding to the terminal points in the curves (fig. S9B).

**Fig. 4.**
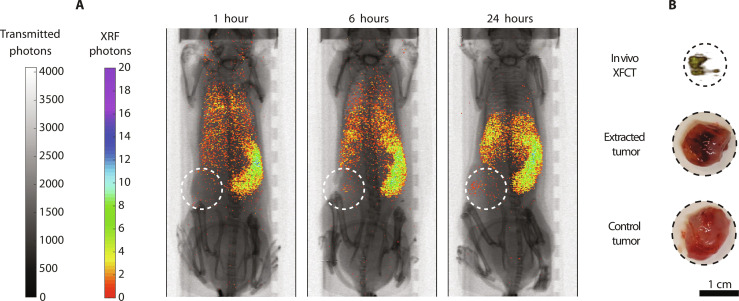
Tumor detection with XRF/XFCT. (**A**) In vivo XRF projection images (1, 6, and 24 hours) of mice injected with clodronate-encapsulated liposomes and MoO_2_-SiO_2_ NPs. The tumor area is highlighted with dashed circles. (**B**) In vivo local XFCT of the tumor 24 hours after NP injection. Pictures of extracted tumors of a NP-injected mouse and a control mouse. Scale bar, 1 cm.

After injecting macrophage-depleted mice with MoO_2_-SiO_2_ NPs, the biodistribution was followed over time, characterized by a gradual NP accumulation process in spleen and, limitedly, in liver, with decreasing scattered signal elsewhere (Fig. 4A and fig. S7). In these mice, Mo XRF signal could be detected within the tumor already 6 hours after NP injection, reaching a peak at 24 hours. A local XFCT in the solid tumor region provided a three-dimensional visualization of the NP distribution within the tumor, with the highest accumulations detected in its core (Fig. 4B). The tumor detection was made possible by the iterative bioengineering process which led to the selection of the combined injection of clodronate-encapsulated liposomes and MoO_2_-SiO_2_ NPs, empowered by XRF imaging. Consistent with previous reports, the enhanced permeability and retention effect alone was not sufficient to detect the tumor (fig. S6), since it typically leads to a small percentage (<1%) of NP delivery to solid tumors (*8*, *33*, *34*).

### Multiscale imaging and nanotoxicology

**Fig. 5.**
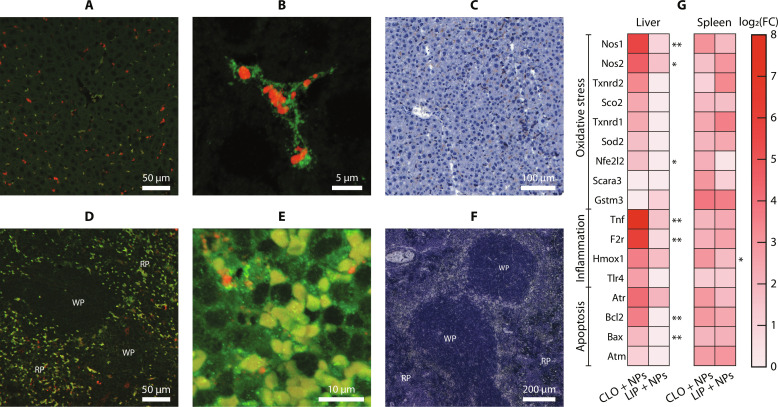
Microscopic and toxicological analysis. Confocal images of liver tissues of a mouse administered with MoO_2_-SiO_2_ NPs and pre-injected with empty liposomes (LIP), using immunofluorescence staining (F4/80 in green, Cy5.5 in red) at (**A**) 10× and (**B**) 63×. (**C**) Microscopy image of liver tissue from the same mouse with chromogenic immunostaining (F4/80, DAB). Confocal images of spleen tissues of a mouse administered with MoO_2_-SiO_2_ NPs and pre-injected with clodronate-encapsulated liposomes (CLO), using immunofluorescence staining (F4/80 in green, Cy5.5 in red) at (**D**) 10× and (**E**) 63×. (**F**) Microscopy image of spleen tissue from the same mouse with chromogenic immunostaining (F4/80, DAB), highlighting red pulp (RP) and white pulp (WP). (**G**) Gene expression quantitative reverse transcription polymerase chain reaction (PCR) in liver and spleen tissues from mice administered with MoO_2_-SiO_2_ NPs, preinjected with CLO or LIP. The intensity is proportional to the log fold change (FC) determined in relation to control tissues. Significant difference between the two groups was indicated when *P < 0.05 or **P < 0.005.

The fluorophore (Cy5.5) present within the silica shell of MoO_2_-SiO_2_ NPs allowed tracking NPs in organ sections by optical fluorescence microscopy, thus offering the possibility to corroborate the XRF biodistribution observations. The high excitation and emission wavelengths of Cy5.5 incorporated in MoO_2_-SiO_2_ NPs (fig. S3B) could minimize the overlap with tissue autofluorescence for postmortem examination (*35*). Immunofluorescence and chromogenic staining of extracted liver and spleen were performed to correlate the XRF imaging distribution with microscopic observations (Fig. 5 and figs. S10 and S11). Confocal images of liver tissues from mice injected with empty liposomes and MoO_2_-SiO_2_ NPs evidenced the phagocytic action of the Kupffer cells. These were stained with an F4/80 antibody recognizing a cell surface glycoprotein expressed in macrophages and a fluorescent conjugate (Alexa Fluor 488). NPs could be localized by Cy5.5 detection within the macrophage cytoplasm (Fig. 5, A and B). The presence of Kupffer cells spread within the hepatic parenchyma was confirmed by chromogenic immunostaining (Fig. 5C).

A clear reduction in macrophage infiltration was evident in liver sections from mice that received clodronate-encapsulated liposomes (fig. S10C), when compared to liver sections from control uninjected mice (fig. S10E) and from mice injected with empty liposomes (fig. S10A). The noticeable reduction in XRF signal detection in the liver between mice pre-injected with empty liposomes (fig. S5) and with clodronate liposomes (fig. S6) reflects on to the limited optical fluorescence (Cy5.5) detection in the latter (fig. S10D).

Immunofluorescence (Fig. 5D and fig. S11) and chromogenic immunostaining (Fig. 5F) of the spleen tissues permitted the localization of the myeloid cells (including splenic macrophages), uniformly distributed in the red pulp, surrounded by reticular cells and lymphocytes (*36*). Several autofluorescing erythrocytes were found in the red pulp (Fig. 5, D and E). Splenic macrophages contained intracytoplasmic erythrocytes, due to the splenic erythrophagocytosis of extravasated and damaged erythrocytes (*37*). Erythrocytes could be clearly distinguished from macrophages by their characteristic shape.

MoO_2_-SiO_2_ NPs could successfully be tracked in the spleen of injected mice, with frequent detection in the splenic red pulp of mice pre-injected with clodronate liposomes (Fig. 5, D and E) and occasional accumulations in the absence of pre-injected clodronate (fig. S11B), in-line with the macroscopical observation made with XRF imaging.

The liver and spleen gene profiling showed that MoO_2_-SiO_2_ NPs induced minor changes compared to uninjected control mice (Fig. 5G and table S1). With clodronate pre-injection, significant fold change increases were measured for genes expressed in the liver, while the gene expression in the spleen was limitedly affected. This observation highlighted an opposite trend to the redistribution of NP accumulations (from liver to spleen).

## DISCUSSION

In the present work, we introduced a methodology for rapid iteration in the preclinical evaluation of contrast agents, using XRF/XFCT as the imaging modality. The sequential NP design led to the optimization of the NP administration protocol, eventually preventing NP sequestration in undesired organs, such as liver and lungs, and consenting to passively deliver NPs to tumor xenografts and diagnose splenomegaly, a tumor-induced biological response. XRF imaging could showcase the NP pharmacokinetics and redistribution in vivo, with the absence of artifacts originating from autofluorescence and without partial detection due to low penetration depth, both constituting a typical limitation when performing optical fluorescence imaging (*38*).

For this study, we used syngeneic mouse models, to preserve an intact immune system. By using these models, it becomes possible to investigate the biodistribution of NPs in an immunocompetent setting. This approach yields more dependable predictions regarding the interactions of nanomedicines within the body (*15*, *39*, *40*).

The first iteration consisted of the study on MoO_2_ NP biodistribution. Despite not observing any behavioral changes or pathological signs in mice, previous histology studies of extracted organs denoted the presence of local thrombi in mice injected with MoO_2_ NPs (*27*), probably related to the presence of NP accumulations in lungs, which were successfully detected by XRF molecular imaging. The possibility to track contrast agents in the lungs with XRF imaging constitutes a major advantage compared to MRI, a standard clinical imaging technique that has though limited applications for lung studies, due to susceptibility artifacts originating at the air-tissue interfaces (*41*, *42*). In this respect, the sensitivity for MoO_2_ NPs was previously estimated to be on the order of 10^−2^ mg/ml (*27*), and multimodal contrast agents evidenced a similar sensitivity for XFCT and contrast-enhanced MRI (*42*).

In an attempt to positively influence the NP biocompatibility and biodistribution, the dye-doped silica shell was introduced, yielding MoO_2_-SiO_2_ NPs. These were demonstrated to be less toxic than the core MoO_2_ NPs in vitro; furthermore, the Cy5.5 dye was embedded in the silica shell to enable optical fluorescence imaging (*18*). Here, we showed evidence of how the silica shell substantially affected the biodistribution with XRF images of mice injected with MoO_2_-SiO_2_ NPs—preceded by an intraperitoneal injection of empty liposome as the control for the subsequent iteration. The migration from the lungs to the liver was ascribed to the cluster nature of the core MoO_2_ NPs, hence being more affected by environmental factors, such as pH and salinity. On the contrary, MoO_2_-SiO_2_ NPs were coated with a uniform layer of amorphous silica, with negatively charged hydroxyl groups preventing further clustering.

NP sequestration by the liver is one of the main biological barriers, accumulating up to 99% of the injected dose and, thus, preventing the delivery of nanomedicines to the desired target (*43*, *44*). Several studies using different kinds of inorganic NPs indicated that these are mostly taken up by hepatic nonparenchymal cells, such as liver macrophages (Kupffer cells) and liver sinusoidal endothelial cells, rather than by the predominant constituents of the liver (parenchymal hepatocytes) (*44*). Furthermore, the flow dynamics of the liver sinusoid favors the NP uptake (*29*). For this reason, the third step in our iterative approach focused on the introduction of clodronate-encapsulated liposomes, aiming at temporarily depleting Kupffer cells (*45*, *46*) before NP injection. Kupffer cells are known to interact with circulating NPs and leading to their sequestration (*47*), and their depletion enabled tumor detection. The spleen constituted the major barrier after macrophage depletion, confirming previous observations with Au NPs and SiO_2_ NPs (*48*). Despite macrophage depletion, NPs with increasing size due to protein corona formation might have been partially retained in the red pulp, undergoing splenic filtration (*49*). In our case, the enhanced sequestration by the spleen might also have occurred due to the tumor-induced splenomegaly.

A multiscale imaging approach empowered the possibility to correlate observations with confocal microscopy to the XRF imaging biodistribution studies, validating the proposed iterative methodology for preclinical studies. Furthermore, to provide a deeper understanding of the NP redistribution effects on the two majorly affected organs (liver and spleen), we quantified the expression of selected genes associated with cellular pathways activated upon NP exposure, such as inflammation, oxidative stress, and apoptosis. The pre-injection of clodronate increased the expression of several tested genes, indicating that the temporary depletion of Kupffer cells may have unbalanced the liver homeostasis, resulting in increased liver toxicity. Noteworthy, gene expression profiling was performed 24 hours after MoO_2_-SiO_2_ NP administration, thus evidencing an acute response phase which probably decays after NP clearance, as previously observed with other NP contrast agents (*50*). A detailed discussion was provided in the Supplementary Materials.

In summary, with this rapid iterative bioengineering process, we successfully investigated the effects of the surface chemistry (MoO_2_ versus MoO_2_-SiO_2_ NPs) and the macrophage depletion on the NP biodistribution, without the need for invasive methods or postmortem evaluation. The high specificity and sensitivity led to the successful detection of Mo XRF photons with submillimeter resolution in several organs, emphasizing the promising purpose of XRF/XFCT within preclinical pharmacokinetic studies for nanomedicine-based formulations.

## MATERIALS AND METHODS

### Materials

Ammonium heptamolybdate [AHM; (NH_4_)_6_Mo_7_O_24_·4H_2_O], PVP (55 kDa), Cy5.5 mono NHS ester (Cy5.5-NHS), (3-aminopropyl) triethoxysilane (APTES; C_9_H_23_NO_3_Si, 99%), triethylamine (TEA; C_6_H_15_N, ≥99%), dimethyl sulfoxide (DMSO; C_2_H_6_OS·H_2_O, ≥99%), tetraethyl orthosilicate (TEOS; C_8_H_20_O_4_Si ≥ 99%), and EA (C_2_H_7_NO, ≥99%) were all purchased from Sigma-Aldrich (Stockholm, Sweden). Ethanol (EtOH) absolute (≥99.8%) was obtained from VWR (Stockholm, Sweden). A MilliQ reference water purification system (Merck Millipore) was used for deionized (DI) water. Clodronate-encapsulated liposomes and empty (control) liposomes were purchased from Liposoma BV (Amsterdam, The Netherlands).

### Core NP synthesis

MoO_2_ NPs were synthesized with a solvothermal method (*18*). The precursor AHM (3.6 mM) was dissolved in 54 ml of DI water and 24 ml of EtOH. PVP (0.29 mM) was added, followed by stirring for 30 min. Using a stainless-steel autoclave with Teflon lining, the synthesis was performed at 180°C for 18 hours (fig. S1A). The obtained MoO_2_ NPs were washed by centrifugation and redispersion in DI water.

### Dye conjugation

Cy5.5-NHS was conjugated with APTES to enable silica doping with a fluorophore during the condensation reaction (*51*). Cy5.5-NHS (1 mg), APTES (0.3 μl), and TEA (0.2 μl) were dispersed in DMSO (50 μl) and left stirring for 24 hours, lastly obtaining Cy5.5-APTES.

### Silica shell condensation

A modified Stöber method with EA as the base was used as previously reported in literature (*18*, *25*), with a few modifications. In a water/EtOH mix (1/3.75), MoO_2_ NPs were dispersed (0.25 mg ml^−1^ in a total of 19 ml). TEOS (10 μl) was introduced, and the dispersion was stirred for 30 min. Cy5.5-APTES (1 μl) and EA (200 μl) were added, continuing stirring (2 hours). The as-obtained MoO_2_-SiO_2_ NPs were washed by centrifuging with EtOH (×1) and water (×1) and lastly dispersed in water and stored at 4°C (fig. S1B).

### Characterization techniques

The surface charges (ζ-potentials) and hydrodynamic [dynamic light scattering (DLS)] sizes were measured in water (pH 6.5) using the Zetasizer Nano ZS90 system (Malvern, UK). Reported DLS size values are number-average values. TEM (JEM-2100F, 200 kV, JEOL) was used to evaluate the morphology and size of dried NPs. Copper grids were used, drop casting 10 μl of the samples and dried at room temperature. For the TEM size analysis, at least 350 NPs/clusters in different field of views were measured. PL (spectrofluorometer, FP-8300, Jasco) was used for the analysis of optical fluorescence properties of the core-shell NPs. The crystallographic phase was determined using SAED in TEM. The presence of PVP and SiO_2_ is confirmed by FTIR (Thermo Fisher Scientific). Limulus Amebocyte Lysate (LAL) assay Endosafe-PTS (Charles River) and PTS cartridges [sensitivity of 0.005 Endotoxin Units (EU) ml^−1^] were used to test the NP stocks for lipopolysaccharides (LPS) contamination. All the stocks used for in vitro and in vivo studies presented LPS values below the maximum admissible limit of 0.1 EU ml^−1^. Mo concentration was estimated with XRF (Mo Kα), by preparing 10× diluted dispersions of MoO_2_ NPs and MoO_2_-SiO_2_ NPs. Mo standard solution [1000 parts per million (ppm)] and water in 2 ml were used as the reference and for background removal, respectively.

### Cytotoxicity assay

To assess the in vitro NP biocompatibility, the real-time cell analysis assay (xCELLigence Agilent, St Clara, USA) assay was performed on RAW264.7 (ATCC-TIB-71) and 4T1 (ATCC-CRL-2539) cell lines, at two concentrations (200 and 100 ppm) in triplicates (96-well plate, biological replicates). Untreated cells were the negative control. The estimated viability was based on the quantification of the impedance, an indicator of cell proliferation. The cells were allowed to adhere to the plate surface for 24 hours before introducing the NPs (time = 0). The signal was normalized to the control cells for each time point (±SD).

### Animal studies

Experiments with mice were approved by the regional animal ethics committee of Northern Stockholm, Sweden (ethical permit number 13156-2022, according to institutional, national, and European guidelines for animal handling and research (L150/SJVFS 2019:9 and 2010/63/EU). Eight-week-old female albino mice (BALB/cAnNRj) were obtained from Janvier Labs (France) and housed under controlled temperature (21° ± 1°C) and humidity (55 ± 5%) conditions, with light-dark cycle and ad libitum feeding. The general conditions of the mice were assessed before and during the study, checking for possible onsets of behavioral and/or morphological changes. Their weight was monitored during the whole study duration, measuring variations lower than 15% (fig. S8B). In a preliminary study, two mice were intravenously injected with MoO_2_ NPs (100 μl, 20 mg/kg) and euthanized after 1 week, to confirm previous results (*27*). For the final study, eight mice were xenografted with the syngeneic breast tumor adenocarcinoma cell line 4T1 by subcutaneously injecting 10^6^ cells suspended in phosphate-buffered saline (PBS; 100 μL). This tumor cell line is characterized by rapid local growth giving palpable tumors of approximately 0.5 cm^3^ within 13 days. The xenografted mice were intraperitoneally injected with either clodronate-encapsulated liposomes (*n* = 4) or empty liposomes (*n* = 4) dispersed in PBS (200 μl, 40 mg/kg). After 72 hours, MoO_2_-SiO_2_ NPs were intravenously injected (100 μl, 30 mg/kg) into these mice.

### XRF imaging

XRF projection images and XFCT scans were acquired in vivo with an imaging arrangement detailed in the Supplementary Materials. Scans were performed under anesthesia with isoflurane (Abbott, Sweden) at several time points. During the imaging sessions, ophthalmic ointment (Oculentum simplex, APL, Sweden) was applied to the eyes for cornea protection; temperature and respiration were also monitored. For whole-body XRF projection imaging, a step size of 200 μm and exposure time of 10 ms per step were chosen, resulting in a 15-min scanning time. A local XFCT was acquired in the tumor area (1-cm vertical region) with a voxel size of 200 μm by 200 μm by 400 μm, acquiring 30 projections over 180°, for a total scanning time of 45 min. The average radiation dose was estimated as 1 and 22 mGy for a whole-body projection image and a local XFCT, respectively (*27*).

### Histological analysis

At the imaging endpoint, the mice were euthanized by carbon dioxide (CO_2_) inhalation. Liver, spleen, and tumor were excised and fixed in 4% buffered paraformaldehyde (PFA) solution for 24 hours, following the protocol for histological preparation (*52*). Briefly, after 24 hours in PFA solution, organs were transferred into 70% ethanol for storing, before vacuum infiltration processing and embedding in paraffin. By using a rotary microtome, 4-μm-thick organ sections were obtained and mounted on standard object glasses. Formaldehyde fixed-paraffin embedded (FFPE) sections were run through automated deparaffinization and rehydration for morphological evaluation. A representative small fresh tissue sample from organs was also collected in TRIzol reagent (Invitrogen, USA) and cryopreserved for RNA isolation before PFA fixation. Immunofluorescence (Alexa Fluor 488) and chromogenic [3,3′-Diaminobenzidine (DAB)] staining of macrophages was performed on FFPE sections, using an F4/80 antibody. Images were obtained using a Zeiss LSM800-Airy (Carl Zeiss GmbH, Germany) with two active laser lines at 488 and 640 nm and emission filters. Two objectives were used for image acquisition (10× air, 63× water). The chromogenic-stained sections were scanned with optical microscopy, and the obtained images were evaluated using the Cytomine web platform (Cytomine Corporation SA Belgium).

### Gene expression analysis

RNA was isolated from liver tissues postmortem using an RNA isolation kit (Zymo Research, California, USA) according to the manufacturer’s instructions. The quality and concentration of the RNA were evaluated by microcapillary electrophoresis using the Agilent Bioanalyser 2100 (Agilent), before cDNA synthesis (AzuraQuant). The expression of genes associated with inflammation, oxidative stress, and apoptosis was evaluated (in triplicates) by quantitative reverse transcription polymerase chain reaction (PCR) using AzuraView GreenFast quantitative PCR reagents (Azura Genomics, USA) and a custom-made PCR array (RealTimePrimers, Elkin Park PA, USA). The tested genes were summarized in table S1. Gene expression was estimated using the Livak method (*53*). The ΔCT values were calculated by normalizing values for each gene to the housekeeping genes, and ΔΔCT was calculated by normalizing the ΔCT values of the NP exposed sample in relation with control untreated sample. The data related to expressed genes were plotted as a function of log fold change, compared to control (uninjected) mice. Significant differences in gene expression in liver and spleen between mice pre-injected with empty liposomes and mice pre-injected with clodronate-encapsulated liposomes were established using the Student’s *t* test.

## Supplementary Materials

### This PDF file includes:

Supplementary Text Figs. S1 to S11 Table S1 References
