# Supplementary material for: Iterative nanoparticle bioengineering enabled by x-ray fluorescence imaging
Source: Sci Adv. 2024 Mar 22;10(12):eadl2267. doi: 10.1126/sciadv.adl2267 (PMC11093098; doi:10.1126/sciadv.adl2267)
Supplement: Supplementary file 1 — Supplementary Text Figs. S1 to S11 Table S1 References [file sciadv.adl2267_sm.pdf]

Science Advances
AAAS

Supplementary Materials for

Iterative nanoparticle bioengineering enabled by x-ray fluorescence imaging

Giovanni M. Saladino *et al.*

Corresponding author: Giovanni M. Saladino, saladino@kth.se

Sci. Adv. **10**, eadl2267 (2024)
DOI: 10.1126/sciadv.adl2267

This PDF file includes:

Supplementary Text
Figs. S1 to S11
Table S1
References

Supplementary Text

Cytotoxicity Studies

The synthesized MoO_2-SiO_2 NPs were evaluated *in vitro* for their cytotoxicity. For this purpose, the real-time cell analysis (RTCA) assay was employed, measuring the cell proliferation over time, normalized to (control) cells unexposed to NPs. RAW 264.7 macrophages were chosen as the first tested cell line as they constitute the main immune response to systemically injected nanomedical formulations, responsible for opsonization. 4T1 breast cancer cell line was selected as they constitute a syngeneic mouse model with xenografted BALB/c mice.

The effects of MoO_2-SiO_2 NPs were concentration, exposure time, and cell line dependent (**Fig. S4A**, **Fig. S4B**). RAW 264.7 macrophages underwent a more acute response during the first 48 h after exposure (IC50 = 200 µg/mL at 24 h). We attributed this response to the macrophages' role in NP phagocytosis through opsonization, constituting a barrier from external offensives (*54*). On the other hand, the 4T1 cell viability never dropped under 75% for either of the two concentrations, within the observation time (72 h).

X-ray Fluorescence Imaging Setup

The XFCT imaging arrangement includes a liquid-metal-jet microfocus source (170 W, 120 kV) and multilayer optics (W-C-coated multilayer Montel mirror). The mirror selects the 24 keV emission (In Kα) from the source, producing a semi-monochromatic high-brightness pencil beam (100×100 µm^2 FWHM focus). For the transmission signal, a 25 mm^2 CdTe detector (X123-CdTe, Amptek Inc., MA) was employed to integrate the whole transmitted spectrum for each pixel, while a 17 mm^2 silicon-drift detector (X123-SDD, Amptek Inc., MA) was used to detect the XRF signal by integrating the spectral range of interest (Mo Kα). The transmission signal was reconstructed using a standard filtered back-projection (FBP) algorithm, while the XRF signal reconstruction was performed using our self-absorption-correcting iterative reconstruction algorithm (*21*).

Contrary to synchrotron beams, the employment of the liquid-metal-jet microfocus source requires the synthesis of NPs containing elements with K-edges close to the fixed source energy (24 keV), leaving a small selection of elements to have sufficient transmission through the tissue. Nevertheless, it has the advantage of portability and, thus, higher accessibility. For our source energy, the estimated XRF tissue transmission is 31% for Mo at 10 mm depth, sufficient for whole-body small-animal XFCT (*27*). While synchrotron sources at high energies (≈ 90 keV) require spatial and spectral filtering schemes for background reduction on human-sized scales (*55*), our lower source energy (24 keV) and smaller samples (≈ 1 cm) only necessitate spectral filtering to remove Compton scattering contributions from Mo Kα emission. This is achieved by integrating only photons within a confined energy range ([17.1 17.7] keV). When using elements with higher Kα emission, multiple Compton scattering background could be reduced by employing focused anti-scatter grids, resulting in the highest overall signal significance (*56*).

Effect of Clodronate in Spleen

The injection of clodronate liposomes did not reduce the macrophage infiltration in spleen, as detected by F4/80 immunofluorescence (**Fig. S11C**), when compared to control mice (**Fig. S11E**). This observation could be correlated with splenomegaly (*57*, *58*), which was successfully detected with XRF imaging. Considering also the reported lower clodronate efficiency in depleting splenic macrophages compared to Kupffer cells (*59*), the injected dose of clodronate liposomes was likely insufficient to effectively deplete splenic macrophages within this altered clinical picture.

Gene Expression Studies

The pre-injection of clodronate liposomes induced oxidative stress in the liver, particularly regarding *Nos1, Nos2*, and *Nfe2l2* expression. *Nos1* and *Nos2* are responsible for nitric oxide formation and important mediators of liver physiology (*60*). *Nfe2l2* is a major regulator of oxidative stress responses in the liver, by expressing antioxidant proteins which protect against oxidative damage (*61*, *62*). Clodronate also induced a pro-inflammatory gene expression profile in liver, mostly characterized by significant increments in the genes *Tnf* and *F2r*. The former exhibited the highest FC ($log_2FC = 7.2$) and is a major player in the inflammation pathways (*63*); transient *Tnf* upregulation has also been observed in liver or rats exposed to gold NPs (*64*), suggesting a specific biological mechanism against nano-sized foreign entities. *F2r* encodes the protease-activated receptor 1 (PAR1) and is expressed in different types of cells (*65*). Two genes associated with apoptosis were upregulated: *Bax* and *Bcl2*. They possess an opposite function in apoptosis (*66*), and their expression ratio has an impact on disease prognosis (*67–69*). The spleen gene expression was limitedly affected by the pre-injection with clodronate-encapsulated liposomes, with only a significant increase in the expression of *Hmox1*. Its activation is mainly localized in the spleen and has the role of facilitating the erythrocyte recycling (*70*).

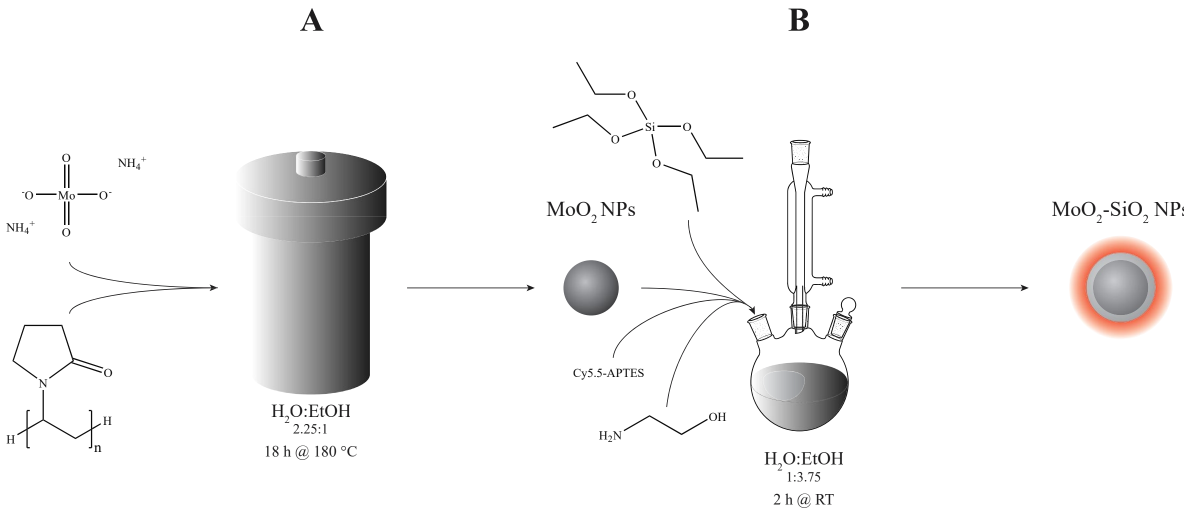

Fig. S1.

Synthesis scheme for MoO_2 NPs and MoO_2-SiO_2 NPs. (**A**) MoO_2 NPs are synthesized with a solvothermal method using AHM and PVP, as the precursor and capping agent, respectively. (**B**) A dye-doped silica (SiO_2) shell is condensed on MoO_2 NPs with a modified Stöber method, using TEOS, Cy5.5-APTES, and EA as the silica precursor, dopant, and base, respectively. The process leads to optically fluorescent MoO_2-SiO_2 NPs.

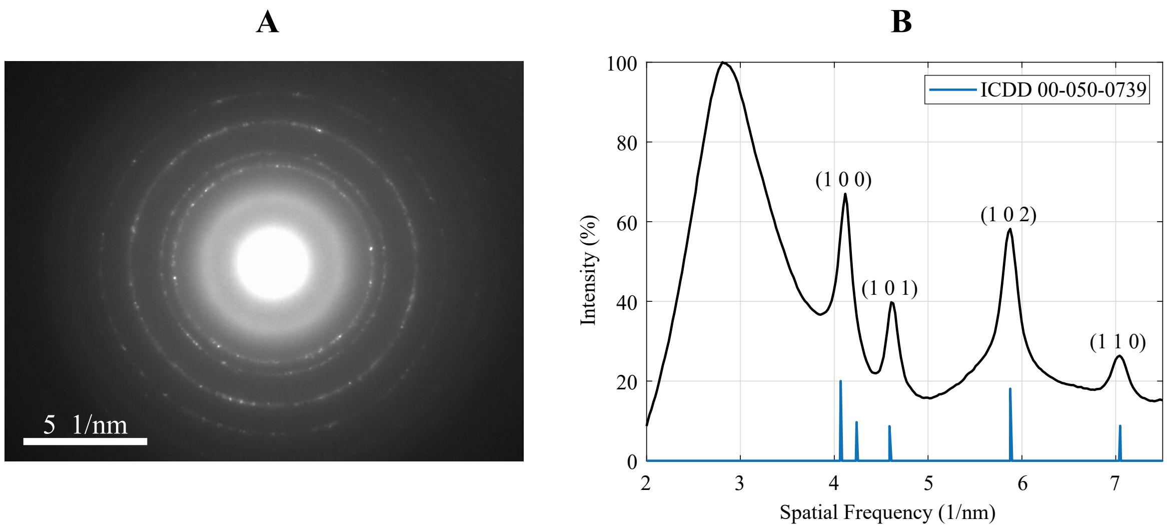

Fig. S2.

Crystallographic analysis. (**A**) SAED pattern for MoO_2-SiO_2 NPs. Scale bar is 5 1/nm. (**B**) 2D profile (black) obtained by circular integration and comparison with standard crystallographic data (ICDD 00-050-0739), in blue. The identified planes are indicated in the plot. The broad peak at low frequency is due to the amorphous silica shell.

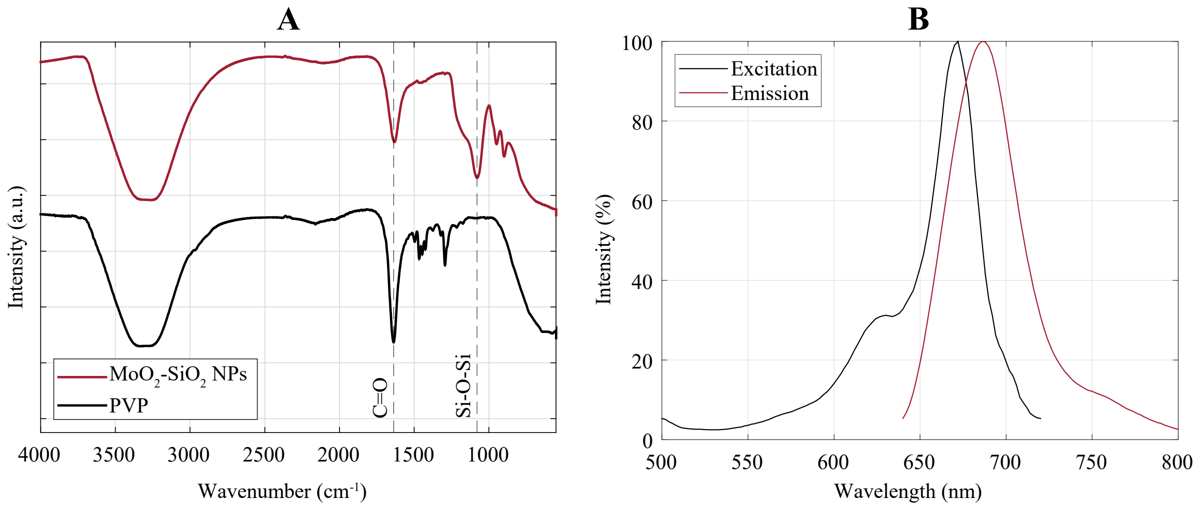

Fig. S3.

Infrared and photoluminescence analysis. (**A**) FT-IR spectra of MoO_2-SiO_2 NPs (red) and PVP (black). The carbonyl (C=O) band from PVP and the Si-O-Si band from the silica condensation reaction are indicated with dashed lines. (**B**) Excitation (black) and emission (red) spectra of the synthesized MoO_2-SiO_2 NPs, with peaks at 672 nm and 686 nm, respectively.

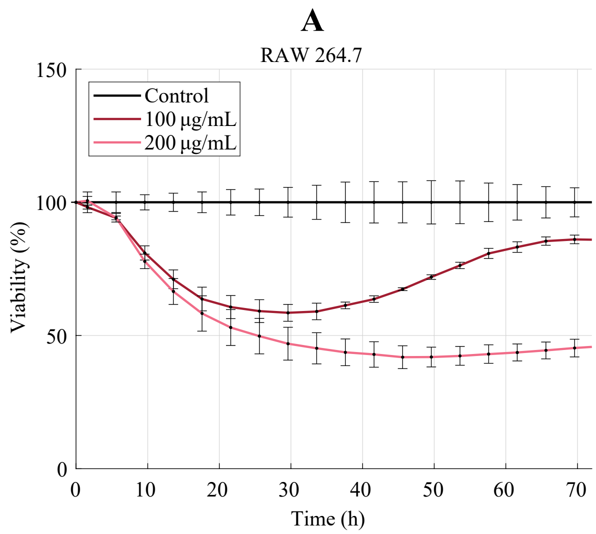

Fig. S4.

Cytotoxicity study. RTCA assay on (**A**) macrophages (RAW 264.7) and (**B**) a breast cancer cell line (4T1), after exposure to MoO_2-SiO_2 NPs ($t \geq 0$). The values at each time point are normalized to unexposed (negative) control cells. Measurements were made in triplicates (± SD).

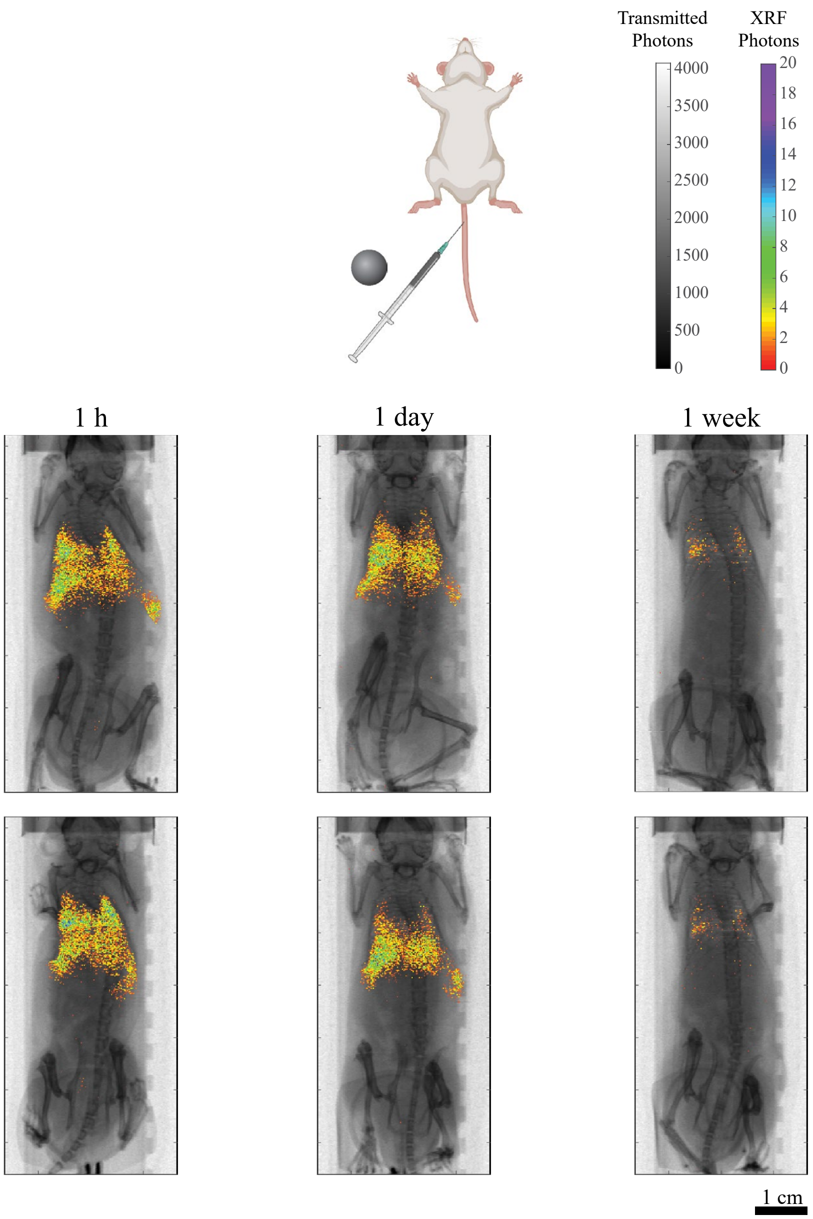

Fig. S5.

XRF imaging with MoO_2 NPs. *In vivo* imaging of mice injected with MoO_2 NPs (1 h, 1 day, and 1 week after NP injection).

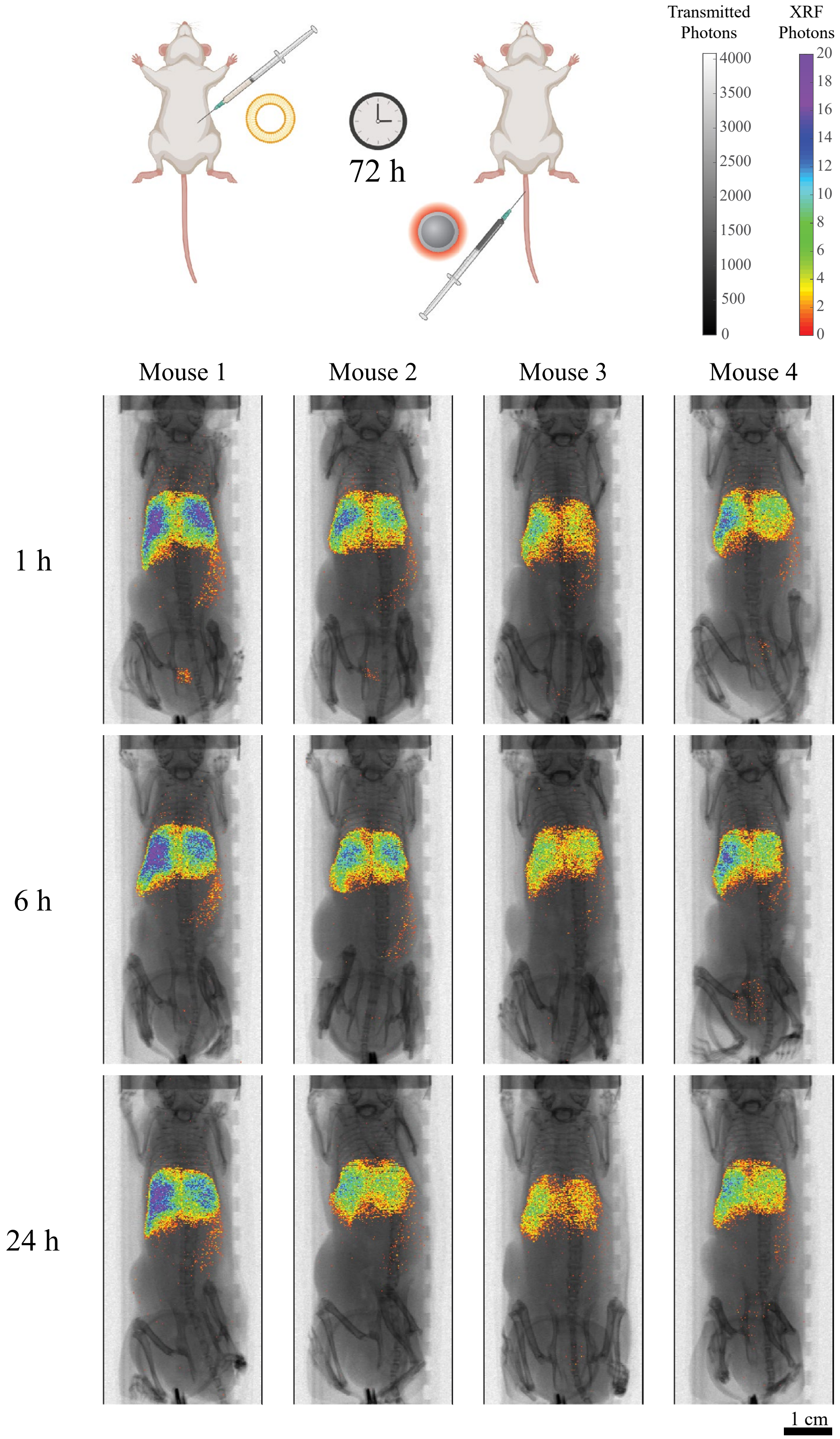

Fig. S6.

XRF imaging with MoO_2-SiO_2 NPs. *In vivo* imaging of mice injected with MoO_2-SiO_2 NPs and pre-injected with empty liposomes 72 h before NP injection.

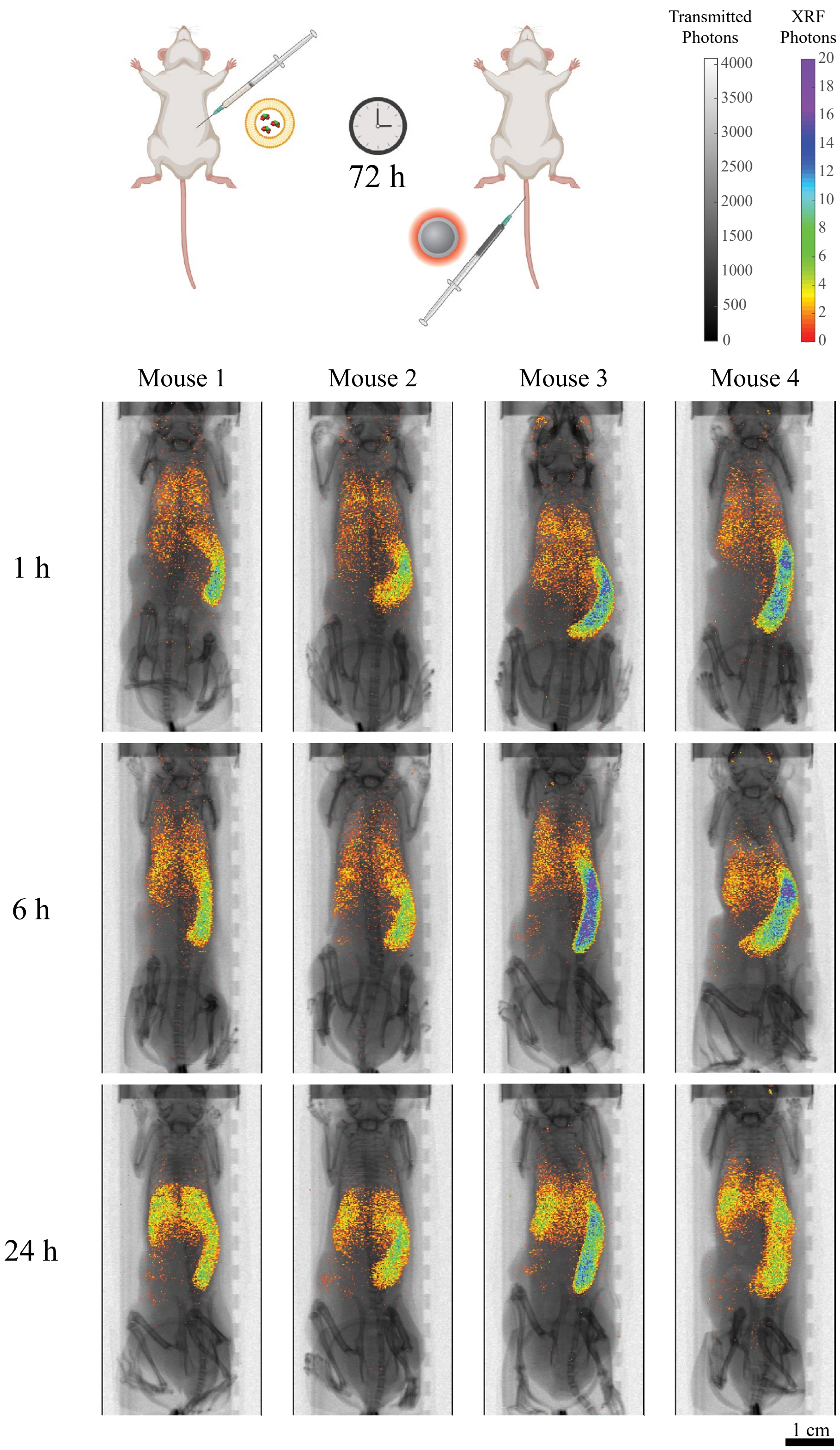

Fig. S7.

XRF imaging with Macrophage Depletion. *In vivo* imaging of mice injected with MoO_2-SiO_2 NPs and pre-injected with clodronate liposomes 72 h before NP injection.

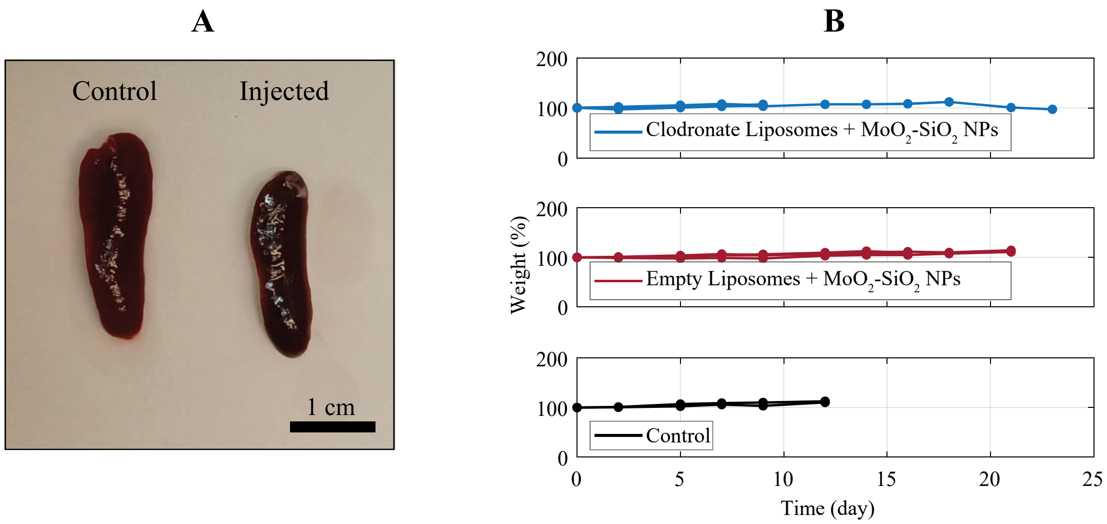

Fig. S8.

Spleen and Weight Monitoring. (**A**) Picture of extracted spleen from an uninjected control mouse (left) and a mouse injected with clodronate-encapsulated liposomes and MoO_2-SiO_2 NPs (right). Scale bar is 1 cm. (**B**) Mouse weight monitoring of the different mouse groups. Values are normalized to the initial mouse weight (≈ 20 g). Variations were lower than 15% during the study.

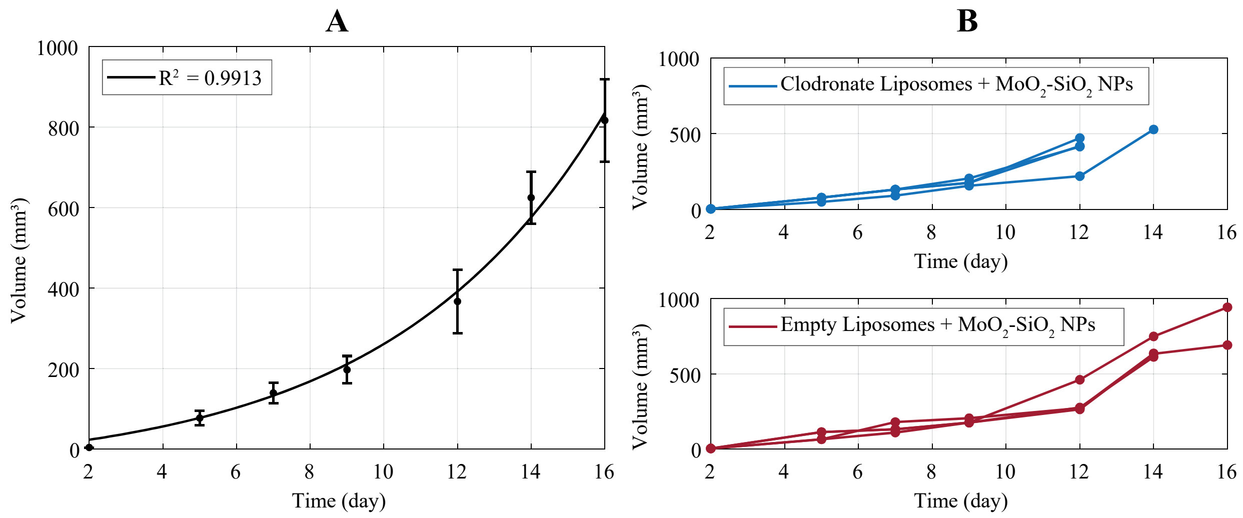

Fig. S9.

Tumor Growth Monitoring. (**A**) Average xenografted tumor volume (± SD) as a function of time. Exponential function was fit to the experimental data (R^2 = 0.9913). (**B**) Tumor volumes for the different injected mouse groups. The terminal point corresponds to injection day.

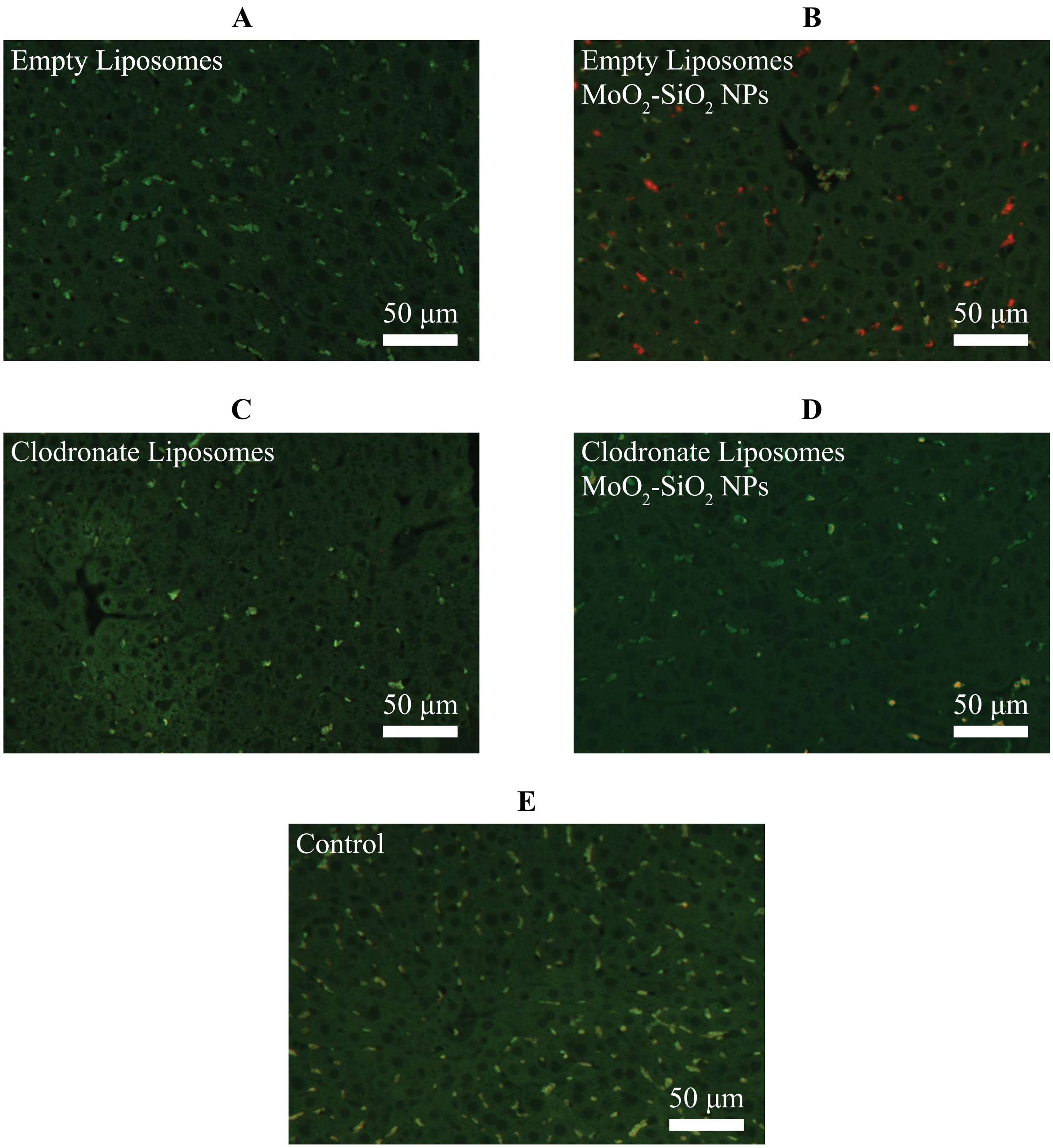

Fig. S10.

Liver Tissue Immunofluorescence. Representative confocal images of liver tissues with immunofluorescence staining (F4/80 in green, Cy5.5 in red, 10×) from different mouse groups: (**A**) empty liposomes (LIP), (**B**) MoO_2-SiO_2 NPs with LIP pre-injection, (**C**) clodronate-encapsulated liposomes (CLO), (**D**) MoO_2-SiO_2 NPs with CLO pre-injection and (**E**) uninjected (control). Scale bars are 50 µm.

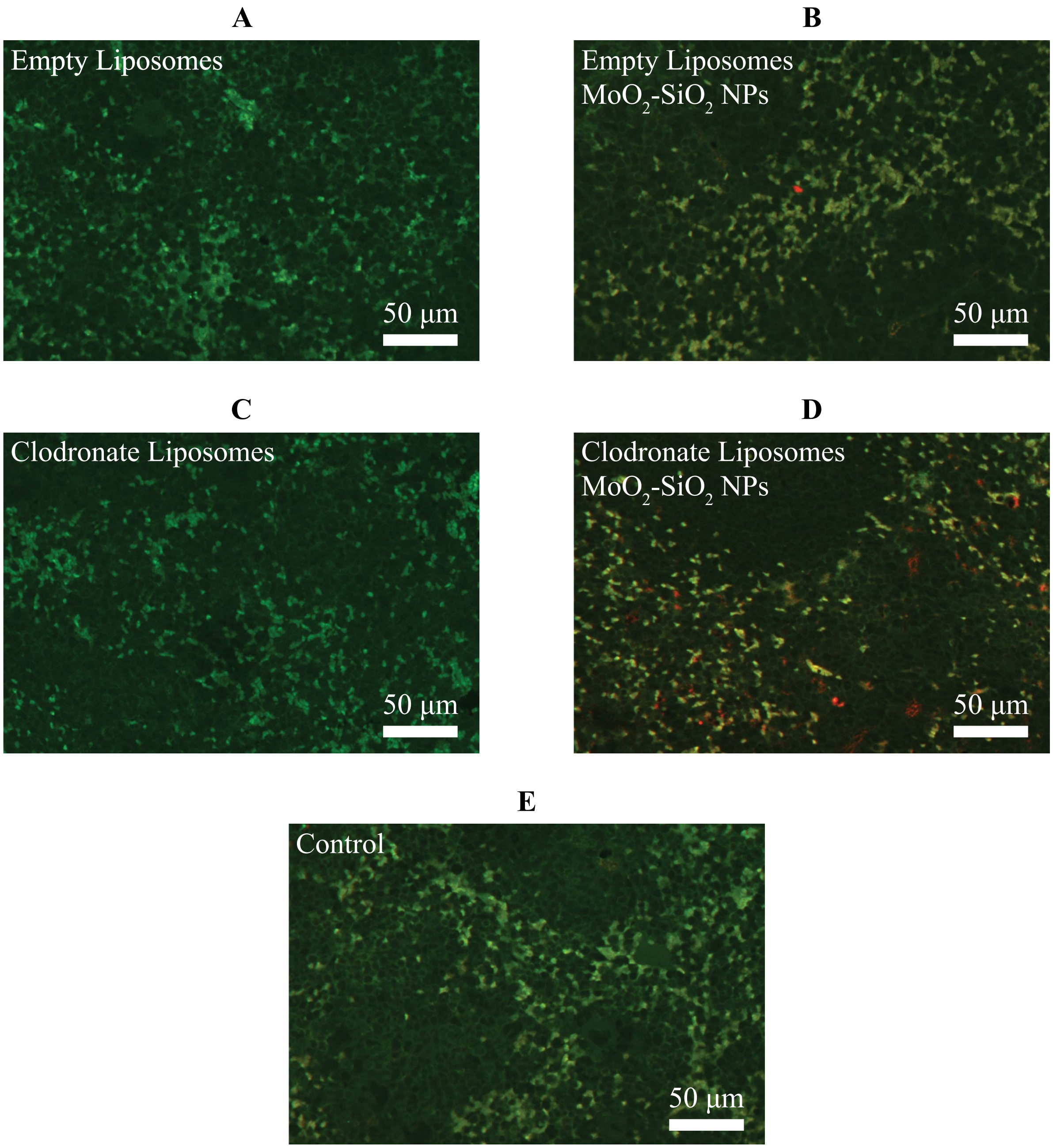

Fig. S11.

Spleen Tissue Immunofluorescence. Representative confocal images of spleen tissues with immunofluorescence staining (F4/80 in green, Cy5.5 in red, 10×) from different mouse groups: (**A**) empty liposomes (LIP), (**B**) MoO_2-SiO_2 NPs with LIP pre-injection, (**C**) clodronate-encapsulated liposomes (CLO), (**D**) MoO_2-SiO_2 NPs with CLO pre-injection and (**E**) uninjected (control). Scale bars are 50 µm.

Table S1.

List of tested genes with qRT-PCR, yielding a toxicity profiling on oxidative stress response, inflammation, and apoptosis. Housekeeping genes were used to calculate the ΔCT values.

**Gene**	**Name**	**Pathway**
Txnrd1	Thioredoxin reductase 1	Oxidative stress
Txnrd2	Thioredoxin reductase 2	Oxidative stress
Sod2	Superoxid dismutase 2 mitochondrial	Oxidative stress
Scara3	Scavenger receptor class A, member 3	Oxidative stress
Nos2	Nitric oxide synthase 2	Oxidative stress
Nos1	Nitric oxide synthase 1	Oxidative stress
Gstm3	Glutathione S-transferase M3	Oxidative stress
Sco2	Cytochrome c oxidase	Oxidative stress
Nfe2l2	Nuclear factor erythroid 2-related factor 2	Oxidative stress
Tnf	Tumour necrosis alpha	Inflammation
Tlr4	Toll-like receptor 4	Inflammation
Hmox1	Heme oxigenase 1	Inflammation
F2r	Coagulation factor II (thrombin) receptor	Inflammation
Bax	BCl2 associated x	Apoptosis
Bcl2	B-cell CLL/lymphoma2	Apoptosis
Atr	Ataxia telangetaxia and RAD 3 related	Apoptosis
Atm	Ataxia telangetaxia	Apoptosis
Gapdh	Glyceraldehyde phosphate dehydrogenase	Housekeeping gene
B2m	Beta-2-microglobulin	Housekeeping gene
Actb	Actin beta	Housekeeping gene

REFERENCES AND NOTES

1. M. J. Mitchell, M. M. Billingsley, R. M. Haley, M. E. Wechsler, N. A. Peppas, R. Langer, Engineering precision nanoparticles for drug delivery. *Nat. Rev. Drug Discov.* **20**, 101–124 (2020).

2. G. Tilstra, J. Couture-Senécal, Y. M. A. Lau, A. M. Manning, D. S. M. Wong, W. W. Janaeska, T. A. Wuraola, J. Pang, O. F. Khan, Iterative design of ionizable lipids for intramuscular mRNA delivery. *J. Am. Chem. Soc.* **145**, 2294–2304 (2023).

3. L. Rodriguez-Lorenzo, S. D. Rafiee, C. Reis, A. Milosevic, T. L. Moore, S. Balog, B. Rothen-Rutishauser, C. Ruegg, A. Petri-Fink, A rational and iterative process for targeted nanoparticle design and validation. *Colloids Surf. B Biointerfaces* **171**, 579–589 (2018).

4. S. M. Park, A. Aalipour, O. Vermesh, J. H. Yu, S. S. Gambhir, Towards clinically translatable in vivo nanodiagnostics. *Nat. Rev. Mater.* **2**, 17014 (2017).

5. A. Cox, P. Andreozzi, R. Dal Magro, F. Fiordaliso, A. Corbelli, L. Talamini, C. Chinello, F. Raimondo, F. Magni, M. Tringali, S. Krol, P. Jacob Silva, F. Stellacci, M. Masserini, F. Re, Evolution of nanoparticle protein corona across the Blood-brain barrier. *ACS Nano* **12**, 7292–7300 (2018).

6. K. Hola, Z. Markova, G. Zoppellaro, J. Tucek, R. Zboril, Tailored functionalization of iron oxide nanoparticles for MRI, drug delivery, magnetic separation and immobilization of biosubstances. *Biotechnol. Adv.* **33**, 1162–1176 (2015).

7. W. Najahi-Missaoui, R. D. Arnold, B. S. Cummings, Safe nanoparticles: Are we there yet?. *Int. J. Mol. Sci.* **22**, 385 (2020).

8. S. Wilhelm, A. J. Tavares, Q. Dai, S. Ohta, J. Audet, H. F. Dvorak, W. C. W. Chan, Analysis of nanoparticle delivery to tumours. *Na. Rev. Mater.* **1**, 16014 (2016).

9. J. W. Shreffler, J. E. Pullan, K. M. Dailey, S. Mallik, A. E. Brooks, Overcoming hurdles in nanoparticle clinical translation: The influence of experimental design and surface modification. *Int. J. Mol. Sci.* **20**, 6056 (2019).

10. J. M. Metselaar, T. Lammers, Challenges in nanomedicine clinical translation. *Drug Deliv. Transl. Res.* **10**, 721–725 (2020).

11. M. R. Junttila, F. J. De Sauvage, Influence of tumour micro-environment heterogeneity on therapeutic response. *Nature* **501**, 346–354 (2013).

12. E. A. Sykes, Q. Dai, C. D. Sarsons, J. Chen, J. V. Rocheleau, D. M. Hwang, G. Zheng, D. T. Cramb, K. D. Rinker, W. C. W. Chan, Tailoring nanoparticle designs to target cancer based on tumor pathophysiology. *Proc. Natl. Acad. Sci. U. S. A.* **113**, E1142–E1151 (2016).

13. S. Goel, C. G. England, F. Chen, W. Cai, Positron emission tomography and Nanotechnology: A dynamic duo for cancer theranostics. *Adv. Drug Deliv. Rev.* **113**, 157–176 (2017).

14. L. Fass, Imaging and cancer: A review. *Mol. Oncol.* **2**, 115–152 (2008).

15. L. Arms, D. W. Smith, J. Flynn, W. Palmer, A. Martin, A. Woldu, S. Hua, Advantages and limitations of current techniques for analyzing the biodistribution of nanoparticles. *Front. Pharmacol.* **9**, 802 (2018).

16. H. M. Hertz, J. C. Larsson, U. Lundström, D. H. Larsson, C. Vogt, Laboratory x-ray fluorescence tomography for high-resolution nanoparticle bio-imaging. *Opt. Lett.* **39**, 2790–2793 (2014).

17. Y. Li, G. M. Saladino, K. Shaker, M. Svenda, C. Vogt, B. Brodin, H. M. Hertz, M. S. Toprak, Synthesis, physicochemical characterization, and cytotoxicity assessment of Rh Nanoparticles with different morphologies-as potential XFCT nanoprobes. *Nanomaterials (Basel)* **10**, 2129 (2020).

18. G. M. Saladino, C. Vogt, Y. Li, K. Shaker, B. Brodin, M. Svenda, H. M. Hertz, M. S. Toprak, Optical and X-ray fluorescent nanoparticles for dual mode bioimaging. *ACS Nano* **15**, 5077 (2021), 5085.

19. G. M. Saladino, N. I. Kilic, B. Brodin, B. Hamawandi, I. Yazgan, H. M. Hertz, M. S. Toprak, Carbon quantum dots conjugated rhodium nanoparticles as hybrid multimodal contrast agents. *Nanomaterials (Basel)* **11**, 2165 (2021).

20. N. I. Kilic, G. M. Saladino, S. Johansson, R. Shen, C. M. Dorman, M. S. Toprak, S. Johansson, Two-Photon Polymerization Printing with High Metal Nanoparticle Loading. *ACS Appl. Mater. Interfaces* **15**, 49794–49804 (2023)

21. J. C. Larsson, C. Vogt, W. Vågberg, M. S. Toprak, J. Dzieran, M. Arsenian-Henriksson, H. M. Hertz, High-spatial-resolution x-ray fluorescence tomography with spectrally matched nanoparticles. *Phys. Med. Biol.* **63**, 164001 (2018).

22. K. N. Clayton, J. W. Salameh, S. T. Wereley, T. L. Kinzer-Ursem, Physical characterization of nanoparticle size and surface modification using particle scattering diffusometry. *Biomicrofluidics* **10**, 054107 (2016).

23. M. Danaei, M. Dehghankhold, S. Ataei, F. Hasanzadeh Davarani, R. Javanmard, A. Dokhani, S. Khorasani, M. R. Mozafari, Impact of particle size and polydispersity index on the clinical applications of lipidic nanocarrier systems. *Pharmaceutics* **10**, 57 (2018).

24. D. Koziej, M. D. Rossell, B. Ludi, A. Hintennach, P. Novák, J. D. Grunwaldt, M. Niederberger, Interplay between size and crystal structure of molybdenum dioxide nanoparticles—synthesis, growth mechanism, and electrochemical performance. *Small* **7**, 377–387 (2011).

25. G. M. Saladino, R. Kakadiya, S. R. Ansari, A. Teleki, M. S. Toprak, Magnetoresponsive fluorescent core–shell nanoclusters for biomedical applications. *Nanoscale Adv.* **5**, 1323–1330 (2023).

26. K. Reczyńska, M. Marszałek, A. Zarzycki, W. Reczyński, K. Kornaus, E. Pamuła, W. Chrzanowski, Superparamagnetic iron oxide nanoparticles modified with silica layers as potential agents for lung cancer treatment. *Nanomaterials* **10**, 1076 (2020).

27. K. Shaker, C. Vogt, Y. Katsu-Jimenez, R. V. Kuiper, K. Andersson, Y. Li, J. C. Larsson, A. Rodriguez-Garcia, M. S. Toprak, M. Arsenian-Henriksson, H. M. Hertz, Longitudinal in-Vivo X-ray fluorescence computed tomography with Molybdenum nanoparticles. *IEEE Trans. Med. Imaging* **39**, 3910–3919 (2020).

28. W. Poon, Y. N. Zhang, B. Ouyang, B. R. Kingston, J. L. Y. Wu, S. Wilhelm, W. C. W. Chan, Elimination pathways of nanoparticles. *ACS Nano* **13**, 5785–5798 (2019).

29. K. M. Tsoi, S. A. Macparland, X. Z. Ma, V. N. Spetzler, J. Echeverri, B. Ouyang, S. M. Fadel, E. A. Sykes, N. Goldaracena, J. M. Kaths, J. B. Conneely, B. A. Alman, M. Selzner, M. A. Ostrowski, O. A. Adeyi, A. Zilman, I. D. McGilvray, W. C. W. Chan, Mechanism of hard-nanomaterial clearance by the liver. *Nat. Mater.* **15**, 1212–1221 (2016).

30. Sally A Du Pre', K. W. Hunter Jr, Murine mammary carcinoma 4T1 induces a leukemoid reaction with splenomegaly association with tumor-derived growth factors. *Exp. Mol. Pathol.* **82**, 12–24 (2007).

31. I. Holen, L. Saleh, P. D. Ottewell, M. A. Lawson, In vivo models used in studies of bone metastases. Bone Cancer: Bone Sarcomas and Bone Metastases - From Bench to Bedside, Academic Press (London), 35–53 (2022).

32. G. Hather, R. Liu, S. Bandi, J. Mettetal, M. Manfredi, W. C. Shyu, J. Donelan, A. Chakravarty, Growth rate analysis and efficient experimental design for tumor xenograft studies, *Cancer Inform.* **13**, 65–72 (2014).

33. Y. Matsumura, H. Maeda, A new concept for macromolecular therapeutics in cancer chemotherapy: Mechanism of tumoritropic accumulation of proteins and the antitumor agent smancs. *Cancer Res.* **46**, 6387–6392 (1986).

34. D. Rosenblum, N. Joshi, W. Tao, J. M. Karp, D. Peer, Progress and challenges towards targeted delivery of cancer therapeutics. *Nat. Commun.* **9**, 1410 (2018).

35. G. Hong, A. L. Antaris, H. Dai, Near-infrared fluorophores for biomedical imaging. *Nat. Biomed. Eng.* **1**, 1–22 (2017).

36. A. J. Mitchell, L. C. Pradel, L. Chasson, N. Van Rooijen, G. E. Grau, N. H. Hunt, G. Chimini, Technical advance: Autofluorescence as a tool for myeloid cell analysis. *J. Leukoc. Biol.* **88**, 597–603 (2010).

37. J. M. Ward, Mann P. C., H. Morishima, Frith C. H., "Thymus, spleen, and lymph nodes" in *Pathology of the mouse*, Maronpot RR, Ed. (Cache River Press, 1999).

38. B. Krajnik, L W Golacki, E. Fiedorczyk, M. Bański, A. Noculak, K M Hołodnik, A. Podhorodecki, Quantitative comparison of luminescence probes for biomedical applications. *Methods Appl. Fluoresc.* **9**, 045001 (2021).

39. N. M. La-Beck, A. A. Gabizon, Nanoparticle interactions with the immune system: Clinical implications for liposome-based cancer chemotherapy. *Front. Immunol.* **8**, 246294 (2017).

40. J. Rios-Doria, N. Durham, L. Wetzel, R. Rothstein, J. Chesebrough, N. Holoweckyj, W. Zhao, C. C. Leow, R. Hollingsworth, Doxil synergizes with cancer immunotherapies to enhance antitumor responses in syngeneic mouse models. *Neoplasia* **17**, 661–670 (2015).

41. M. Wielpütz, H. U. Kauczor, MRI of the lung: State of the art. *Diagn. Interv. Radiol.* **18**, 344–353 (2012).

42. G. M. Saladino, C. Vogt, B. Brodin, K. Shaker, N. I. Kilic, K. Andersson, M. Arsenian-Henriksson, M. S. Toprak, H. Hertz, XFCT-MRI hybrid multimodal contrast agents for complementary imaging. *Nanoscale* **15**, 2214–2222 (2023).

43. Y. N. Zhang, W. Poon, A. J. Tavares, I. D. McGilvray, W. C. W. Chan, Nanoparticle–liver interactions: Cellular uptake and hepatobiliary elimination. *J. Control. Release* **240**, 332–348 (2016).

44. J. Li, C. Chen, T. Xia, Understanding nanomaterial–liver interactions to facilitate the development of safer nanoapplications. *Adv. Mater.* **34**, e2106456 (2022).

45. N. Van Rooijen, A. Sanders, Liposome mediated depletion of macrophages: Mechanism of action, preparation of liposomes and applications. *J. Immunol. Methods* **174**, 83–93 (1994).

46. N. Van Rooijen, A. Sanders, T. K. Van Den Berg, Apoptosis of macrophages induced by liposome-mediated intracellular delivery of clodronate and propamidine. *J. Immunol. Methods* **193**, 93–99 (1996).

47. W. Ngo, S. Ahmed, C. Blackadar, B. Bussin, Q. Ji, S. M. Mladjenovic, Z. Sepahi, W. C. W. Chan, Why nanoparticles prefer liver macrophage cell uptake in vivo. *Adv. Drug Deliv. Rev.* **185**, 114238 (2022).

48. A. J. Tavares, W. Poon, Y. N. Zhang, Q. Dai, R. Besla, D. Ding, B. Ouyang, A. Li, J. Chen, G. Zheng, C. Robbins, W. C. W. Chan, C. J. Murphy, Effect of removing Kupffer cells on nanoparticle tumor delivery. *Proc. Natl. Acad. Sci. U. S. A.* **114**, E10871–E10880 (2017).

49. M. Cataldi, C. Vigliotti, T. Mosca, M. R. Cammarota, D. Capone, Emerging role of the spleen in the pharmacokinetics of monoclonal antibodies, nanoparticles and exosomes. *Int. J. Mol. Sci.* **18**, 1249 (2017).

50. C. Vogt, G. M. Saladino, K. Shaker, M. Arsenian-Henriksson, H. M. Hertz, M. S. Toprak, B. A. Brodin, Organ uptake, toxicity and skin clearance of ruthenium contrast agents monitored in vivo by x-ray fluorescence. *Nanomedicine (Lond.)* **18**, 1161–1173 (2023).

51. Y. Lian, L.-J. Ding, W. Zhang, X. Zhang, Y.-L. Zhang, Z. Lin, X. Wang, Synthesis of highly stable cyanine-dye-doped silica nanoparticle for biological applications. *Methods Appl Fluoresc.* **6**, 034002 (2018).

52. C. Antal, M. Teletin, O. Wendling, M. Dgheem, J. Auwerx, M. Mark, Tissue collection for systematic phenotyping in the mouse. *Curr. Protoc. Mol. Biol.* **80**, 4 (2007).

53. K. J. Livak, T. D. Schmittgen, Analysis of relative gene expression data using real-time quantitative PCR and the 2−ΔΔCT method. *Methods* **25**, 402–408 (2001).

54. T. A. Wynn, A. Chawla, J. W. Pollard, Macrophage biology in development, homeostasis and disease. *Nature* **496**, 445–455 (2013).

55. F. Grüner, F. Blumendorf, O. Schmutzler, T. Staufer, M. Bradbury, U. Wiesner, T. Rosentreter, G. Loers, D. Lutz, B. Richter, M. Fischer, F. Schulz, S. Steiner, M. Warmer, A. Burkhardt, A. Meents, M. Kupinski, C. Hoeschen, Localising functionalised gold-nanoparticles in murine spinal cords by X-ray fluorescence imaging and background-reduction through spatial filtering for human-sized objects. *Sci. Rep.* **8**, 16561 (2018).

56. J. C. Larsson, K. Shaker, H. M. Hertz, Focused anti-scatter grid for background reduction in x-ray fluorescence tomography. *Opt. Lett.* **43**, 2591–2594 (2018).

57. B. J. Chernak, R. K. Rampal, Extramedullary hematopoiesis in myeloproliferative neoplasms: Pathophysiology and treatment strategies. *Int. Rev. Cell Mol. Biol.* **365**, 97–116 (2021).

58. E. D. Bührer, M. A. Amrein, S. Forster, S. Isringhausen, C. M. Schürch, S. S. Bhate, T. Brodie, J. Zindel, D. Stroka, M. A. Sayed, C. Nombela-Arrieta, R. Radpour, C. Riether, A. F. Ochsenbein, Splenic red pulp macrophages provide a niche for CML stem cells and induce therapy resistance. *Leukemia* **36** (11), 2634–2646 (2022).

59. G. Schiedner, S. Hertel, M. Johnston, V. Dries, N. van Rooijen, S. Kochanek, Selective depletion or blockade of kupffer cells leads to enhanced and prolonged hepatic transgene expression using high-capacity adenoviral vectors. *Mol. Ther.* **7**, 35–43 (2003).

60. Y. Iwakiri, M. Y. Kim, Nitric oxide in liver diseases. *Trends Pharmacol. Sci.* **36**, 524–536 (2015).

61. S. A. Chanas, Q. Jiang, M. M. Mahon, G. K. Mc Walter, L. I. Mc Lellan, C. R. Elcombe, C. J. Henderson, C Roland Wolf, G. J. Moffat, K. Itoh, M. Yamamoto, J. D. Hayes, Loss of the Nrf2 transcription factor causes a marked reduction in constitutive and inducible expression of the glutathione S-transferase Gsta1, Gsta2, Gstm1, Gstm2, Gstm3 and Gstm4 genes in the livers of male and female mice. *Biochem. J.* **365**, 405–416 (2002).

62. E. Thunnissen, E. F. Smit, Nonsmall-cell cancers of the lung: Pathology and genetics. *Encyclopedia of Cancer*, **76–87** (2018).

63. N. Parameswaran, S. Patial, Tumor necrosis factor-α signaling in macrophages. *Crit. Rev. Eukaryot. Gene Expr.* **20**, 87–103 (2010).

64. H. A. Khan, M. A. K. Abdelhalim, A. S. Alhomida, M. S. Al Ayed, Transient increase in IL-1β, IL-6 and TNF-α gene expression in rat liver exposed to gold nanoparticles. *Genet. Mol. Res.* **12**, 5851–5857 (2013).

65. X. Liu, J. Yu, S. Song, X. Yue, Q. Li, Protease-activated receptor-1 (PAR-1): A promising molecular target for cancer. *Oncotarget* **8**, 107334–107345 (2017).

66. Q. Wang, L. Zhang, X. Yuan, Y. Ou, X. Zhu, Z. Cheng, P. Zhang, X. Wu, Y. Meng, L. Zhang, The Relationship between the Bcl-2/Bax proteins and the mitochondria-mediated apoptosis pathway in the differentiation of adipose-derived stromal cells into neurons. *PLoS One* **11**, e0163327 (2016).

67. K. J. Campbell, S. W. G. Tait, Targeting BCL-2 regulated apoptosis in cancer. *Open Biol.* **8**, 180002 (2018).

68. S. Salakou, A. C. Tsamandas, D. S. Bonikos, T. Papapetropoulos, D. Dougenis, The potential role of bcl-2, bax, and Ki67 expression in thymus of patients with myasthenia gravis, and their correlation with clinicopathologic parameters. *Eur. J. Cardiothorac. Surg.* **20**, 712–721 (2001).

69. A. M. Sharifi, F. E. Hoda, A. M. Noor, Studying the effect of LPS on cytotoxicity and apoptosis in PC12 neuronal cells: Role of Bax, Bcl-2, and Caspase-3 protein expression. *Toxicol. Mech. Methods* **20**, 316–320 (2010).

70. P. Slusarczyk, P. K. Mandal, G. Zurawska, M. Niklewicz, K. Chouhan, R. Mahadeva, A. Jończy, M. Macias, A. Szybinska, M. Cybulska-Lubak, O. Krawczyk, S. Herman, M. Mikula, R. Serwa, M. Lenartowicz, W. Pokrzywa, K. Mleczko-Sanecka, Impaired iron recycling from erythrocytes is an early hallmark of aging. *eLife* **12**, e79196 (2023).
